# Acquisition of Conditioning between Methamphetamine and Cues in Healthy Humans

**DOI:** 10.1371/journal.pone.0161541

**Published:** 2016-08-22

**Authors:** Joel S. Cavallo, Leah M. Mayo, Harriet de Wit

**Affiliations:** 1 Department of Psychiatry and Behavioral Neuroscience, University of Chicago, 5841 S. Maryland Ave, MC3077, Chicago, IL 60637, United States of America; 2 Center for Social and Affective Neuroscience, Department of Clinical and Experimental Medicine, Linkӧping University, SE 58183, Linkӧping, Sweden; Johns Hopkins School of Medicine, UNITED STATES

## Abstract

Environmental stimuli repeatedly paired with drugs of abuse can elicit conditioned responses that are thought to promote future drug seeking. We recently showed that healthy volunteers acquired conditioned responses to auditory and visual stimuli after just two pairings with methamphetamine (MA, 20 mg, oral). This study extended these findings by systematically varying the number of drug-stimuli pairings. We expected that more pairings would result in stronger conditioning. Three groups of healthy adults were randomly assigned to receive 1, 2 or 4 pairings (Groups P1, P2 and P4, Ns = 13, 16, 16, respectively) of an auditory-visual stimulus with MA, and another stimulus with placebo (PBO). Drug-cue pairings were administered in an alternating, counterbalanced order, under double-blind conditions, during 4 hr sessions. MA produced prototypic subjective effects (mood, ratings of drug effects) and alterations in physiology (heart rate, blood pressure). Although subjects did not exhibit increased behavioral preference for, or emotional reactivity to, the MA-paired cue after conditioning, they did exhibit an increase in attentional bias (initial gaze) toward the drug-paired stimulus. Further, subjects who had four pairings reported “liking” the MA-paired cue more than the PBO cue after conditioning. Thus, the number of drug-stimulus pairings, varying from one to four, had only modest effects on the strength of conditioned responses. Further studies investigating the parameters under which drug conditioning occurs will help to identify risk factors for developing drug abuse, and provide new treatment strategies.

## Introduction

Environmental cues that are repeatedly paired with drugs acquire the ability to produce conditioned responses through the process of associative learning ([1–3], see [4–7] for reviews). Drug-associated cues can elicit a multitude of responses thought to be important for the development and persistence of addiction, such as drug craving [8–10], physiological arousal [11], emotional reactivity [12, 13], and attentional bias [14, 15]. These cue-elicited responses are believed to facilitate drug seeking behavior in drug-dependent populations (see [16] for review), and elicit relapse in recently abstinent drug users [17, 18]. Conditioned drug responses have been demonstrated in human drug users for several drugs, including methamphetamine [19], nicotine [20], alcohol [21], and cocaine ([22], see [10, 23, 24] for reviews). Identifying the factors that promote the development of associative learning are of great clinical importance in understanding addictions.

One factor thought to contribute to the strength of conditioning is the number of conditioning trials [25, 26]. In rodents tested in the conditioned place preference procedure, more pairings between cocaine and one context result in place preferences that are stronger or longer lasting ([27–30], but see [31, 32]). The relationship between the number of pairings and conditioned response strength has also been observed in humans using cues paired with either food [33–36] or drug rewards [37, 38]. For example, in non-dependent daily cigarette smokers, more pairings between a cue and smoking resulted in a greater urge to smoke and larger changes in physiological measures in response to the cue [37]. Similarly, Mucha et al (1998) found that in non-dependent daily cigarette smokers, multiple training sessions between neutral tones paired with smoking cigarettes resulted in greater preference for the tones than fewer training sessions [38]. Although the positive relationship between conditioning amount and cue response magnitude is intuitive, it is not always observed. Both Thewissen et al (2006) and Dols et al (2000) used a *de novo* conditioning procedure with non-dependent daily smokers and found that cue-elicited urge to smoke was similar after three or six trials [2, 39]. Clearly, the conditions under which Pavlovian conditioning develops in humans are not fully understood.

Most of the studies conducted to date with drug conditioning in humans involve participants with established histories of drug use. However, with these populations, researchers have little control over participants’ previous drug exposures, their associated cues and contexts, or the memories related to the drug using experience [40]. Studies with healthy volunteers address some of these problems. Thus, our lab has developed a paradigm to study the acquisition of *de novo* drug-cue associations in healthy young adults [3, 41]. This procedure involves pairing neutral audio-visual cues with either methamphetamine (MA; 20 mg oral) or placebo (PBO), two times each. Conditioning is measured with assessments of behavioral preference, attentional bias, emotional reactivity towards the MA-paired cue, and subjective “liking” of the cue. In the previous study [41], conditioned responses were detected on all measures except cue “liking.” In the present study, we examined the effect of varying the number of drug-cue pairings (1, 2, and 4 pairings) in three groups of participants (P1, P2, and P4, respectively). We tested their responses to the MA- and PBO-paired cues immediately after conditioning and again 2, 7, and 20 days later. We hypothesized that the number of pairings would predict the strength and persistence of conditioning on measures of behavioral preference, attentional bias, and emotional reactivity (relative to the PBO-paired cue). Based on previous observations, we also predicted that some of the conditioned responses would be related to the subjective drug effects during the conditioning phase.

## Materials and Methods

### Design

This study used a between-subjects design consisting of three groups of subjects who received different numbers of pairings of methamphetamine (MA, 20 mg oral) and placebo, each with distinctive stimuli. Group P1 received one pairing of MA and placebo (i.e., 2 sessions), Group P2 received two pairings (4 sessions) and Group P4 received four pairings (i.e., 8 sessions). The study consisted of four phases: i) a pre-test assessing responses to the cues before conditioning, ii) a conditioning phase consisting of one, two, or four presentations of MA, and placebo paired with distinctive audio/visual stimuli, iii) a post-conditioning test, and iv) three online follow-up tests. The primary outcome measures were four indices of conditioning of the MA-paired stimuli: behavioral preference, attentional bias, emotional reactivity, and ‘liking’ of the stimuli. The primary goal was to determine whether the strength or persistence of conditioned responses was related to the number of pairings. The procedure was based on previous studies in our laboratory [3, 41], with minor methodological changes, noted below. This study and written consent forms were approved by the University of Chicago Biological Science Division Institutional Review Board.

### Participants

Healthy volunteers (N = 45) aged 18–35 were recruited from the university and surrounding community using flyers and online advertisements approved by the University of Chicago Institutional Review Board (Table 1). They were screened with a psychiatric interview, electrocardiogram, and physical examination. Inclusion criteria were: BMI of 19–26 kg/m, a high school education, fluency in English, resting blood pressure less than 140/90 mm Hg, resting heart rate less than 90 bpm, and consumption of less than four standard caffeinated or alcoholic beverages per day. Exclusion criteria included: current medications (except hormonal birth control for women), current or past year substance dependence, history of cardiovascular illness, current or last 5 year diagnosis of attention deficit hyperactivity disorder (ADHD), current or past year major Axis I DSM-IV disorder [42], mood disorders, or psychotic symptoms within the past year. Shift workers, pregnant women and nursing mothers were also excluded. Women who were not on hormonal birth control completed all conditioning sessions during the follicular phase of their menstrual cycle [43].

**Table 1:** Participant Demographics and Current/Lifetime Drug Use, Percent (N) or Mean (SEM).

Category		P1 (N = 13)	P2 (N = 16)	P4 (N = 16)
Gender	Male/Female	8/5	8/8	9/7
Race				
	Caucasian	69.2% (9)	62.5% (10)	56.3% (9)
	African-Am.	7.7% (1)	18.8% (3)	25.0% (4)
	Asian	15.4% (2)	6.3% (1)	6.3% (1)
	Other	7.7% (1)	12.5% (2)	12.5% (2)
Age (years)		24.85 (0.83)	25.12 (0.89)	24.88 (0.93)
Education		15.85 (0.42)	16.00 (0.45)	15.25 (0.36)
BMI		23.25 (0.58)	23.11 (0.43)	23.26 (0.43)
Current Drug Use				
	Caffeine, servings/day	1.51 (0.27)	1.72 (0.14)	1.06 (0.20)
	Cigarettes/week	1.32 (1.08)	2.13 (0.99)	5.58 (4.36)
	Alcoholic drinks/week	7.73 (1.79)	7.11 (1.14)	6.11 (1.20)
	Marijuana use, last 30 d	6.08 (2.99)	3.5 (1.31)	4.53 (3.71)
Lifetime Drug Use				
	Marijuana	92.3% (12)	93.8% (15)	81.3% (13)
	Opiates	30.8% (4)	31.3% (5)	25.0% (4)
	Stimulants	46.2% (6)	37.5% (6)	25.0% (4)
	Hallucinogens	30.8% (4)	37.5% (6)	50.0% (8)
	MDMA	23.1% (3)	43.8% (7)	31.3% (5)
	Sedatives	23.1% (3)	12.5% (2)	12.5% (2)

### Drug

Methamphetamine (MA; 20 mg oral; Desoxyn, Lundbeck) dose and dosage procedures were identical to those used previously [3, 41]. Methamphetamine was used because it reliably produces feelings of well-being in healthy young adults [44, 45]. MA tablets were crushed and mixed into 10 ml of equal parts OraSweet and OraPlus syrup (Paddock Laboratories, Minneapolis, MN). The placebo (PBO) consisted of 5 ml OraSweet and 5 ml OraPlus syrups alone.

### Session procedures

#### Orientation Session

During an initial orientation visit, study procedures were explained to participants and written informed consent was obtained. Participants were told that they could be given a placebo, stimulant, sedative or alcohol in order to minimize expectancy effects. Participants were asked to abstain from recreational drug use for 48 h before any session (7 d for marijuana) as well as 6 h after any session, and to consume their normal amounts of caffeine and nicotine before all sessions. Participants practiced the study tasks and questionnaires.

#### All Sessions

Sessions were held in comfortably furnished rooms that contained a television, VHS player, and computer. At each session, participants first completed drug screening and pregnancy tests, subjective drug effects ratings (POMS, ARCI, and DEQ, see below), and cardiovascular measures (blood pressure [BP] and heart rate [HR]). Compliance was assessed at each session using breathalyzers (measuring blood-alcohol levels; Alco-SensorIII, Intoximeters, St Louis, MO), urine drug tests (ToxCup, Branan Medical Corporation, Irvine, CA), and pregnancy tests for women (AimStickPBD, hCG professional, Craig Medical Distribution, Vista, CA). When not completing experimental tasks, participants could watch selected movies, read, or relax.

#### Pre-Test (Session 1)

This 2-hr session was conducted between 9 am and 5 pm. The primary goal was to assess subjects’ baseline responses toward the two to-be-conditioned visual/auditory cues as in [41]. After initial compliance tests, participants viewed images of an ocean accompanied by wave sounds, and a mountain scene accompanied by the sound of birds chirping. Four tasks (described in detail below) were used to assess their pre-conditioning responses to these stimuli: 1) a forced choice task to measure behavioral preference, 2) a modified visual-probe task to track eye gaze via electrooculography (EOG) was used to measure attentional bias, 3) a visual analog scale (VAS) to assess subjective “liking” of the two stimuli, and 4) facial electromyography (EMG) to assess emotional reactivity toward the two cues. See outcome measures below for further detail. The tasks and stimuli were presented with E-Prime 2.0 (PST, Pittsburgh, PA).

#### Conditioning Sessions (Sessions 2–3, 2–5, or 2–9)

These 4-hr sessions were conducted from 9 am to 1 pm, 2–10 d apart. Subjective drug effects and cardiovascular measures were taken six times throughout the session (15 mins before and 15, 30, 70, 115, and 210 mins after drug administration). At 9:30 am, the drug (MA) or placebo (PBO) was administered orally under double blind conditions. Subjects received MA and PBO on alternating sessions, with the order (MA or PBO first) counterbalanced between subjects. Thirty min after MA/PBO administration, timed to coincide with peak drug effects, see [41], participants viewed the stimuli presented on a computer screen. While the cues were displayed, subjects also performed four distractor computer tasks to keep their attention on the computer screen. The visual conditioning stimuli (ocean or mountain) were presented in the background, and occupied about 2/3 of the screen, while the tasks were presented in the middle 1/3 of the screen. The tasks were not used as outcome measures. One conditioning stimulus (ocean or mountain image/sound) was present only when subjects received MA, while the other was only present during PBO sessions. Cue assignments were randomized and counterbalanced between subjects. Participants were allowed to leave at 1 pm if they no longer felt drug effects and their BP/HR measurements had returned to within 20% of baseline values.

The four ‘distractor’ tasks included the Balloon Analogue Risk Taking (BART) task [46], a simple Reaction Time (RT) task [47], the Go/No-go (GNG) task adapted from [48], and the Gluck task [49]. The BART is a measure of risk taking behavior in which subjects inflate virtual balloons to earn points. The RT task is a simple reaction time task in which participants press a button as quickly as possible when a stimulus appears on the screen. The Go/No-go task is a measure of impulsive action in which subjects respond when given a “go” signal, but inhibit responses when a “no-go” signal is given. The Gluck is a probabilistic classification task in which participants win or lose points as they learn to classify arbitrary stimuli. The specific tasks used to sustain subjects’ attention in this study differed from the tasks used in [41]. However, in both cases the tasks required attention and motivated behaviors, and participants were told they could earn points or money based on performance. In the present study, subjects received $5 for the tasks regardless of their performance, although they were told that their payment would be related to their performance. The four tasks presented during stimulus viewing lasted about 30 min, and were presented in a random order between subjects, but held constant within subjects. Tasks were presented using E-Prime 2.0 software.

#### Post-Test (Sessions 4, 6, or 10)

This 2-hr session was conducted at the same time of day as session 1, 2–10 d after the last conditioning session, to assess the change in responses to the conditioning stimuli. Subjects completed the same four tasks conducted during the pre-test: forced choice, visual probe, VAS, and EMG.

#### Follow-Up

Participants also completed online versions of three study tasks (forced choice, visual probe, and VAS) at 2, 7, and 20 d following the post-test session to assess the persistence of the conditioned responses. Participants were asked to use a personal computer to access the tasks on a website using a web application (Inquisit, Millisecond Software Seattle, WA). Participants were instructed to complete the follow-up tasks in a quiet location with the computer audio turned on. The tasks were presented similarly to the pre- and post-test sessions, e.g., stimulus duration, inter-trial interval, etc.

### Outcome Measures

#### Behavioral Preference (Forced Choice Task)

A forced choice task was used to assess behavioral preference for the two cues. Participants viewed composite images of the background scenes with images of distractor tasks superimposed (e.g., ocean + distractor task 1; mountain + distractor task 1). These were presented first individually and then in pairs. Thus, in each trial, two composite pictures were presented individually for 3 s (with audio corresponding to the presented background image), and then the two images were presented side by side (no audio) for the preference test. Participants indicated their preference by pressing the corresponding mouse button (left or right). A total of 28 pairs of background (2 types) plus task images (4 types) were presented using a full-factorial design, and order presentation was randomized. The primary outcome measure was choice preference for the drug-paired cue, which was calculated as the number of trials on which subjects chose the MA-paired background cue when both cues were present on-screen (i.e., the backgrounds differed), minus the number of choices for the PBO-paired backgrounds. This left a total of 16 comparisons of interest. Individual choice behavior was used to calculate the change in preference for the drug-paired cue before and after conditioning. For the purposes of comparison, the prior study using this protocol [41] utilized only three distractor tasks instead of four, and so the present study had a higher number of comparisons. We reasoned that increasing the number of comparisons in the preference scale might increase its sensitivity. To rule out preference biases for specific tasks either before and after conditioning we also examined preference for distractor task images.

#### Self-reported “Liking” (Visual Analog Scale, VAS)

A VAS scale was used as a self-reported measure of subjective “liking” for each cue, and was given during the pretest, post-test, and follow-ups. In this task, the two conditioned stimuli were presented individually, twice for 5 s each. Participants were asked to move a small vertical bar on a horizontal line indicating how much they liked each cue on a scale of 0 (“dislike very much”) to 100 (“like very much”).

#### Attentional Bias (Modified Visual-Probe Task)

Bias in attention towards the two conditioned stimuli was examined using a modified visual-probe task in combination with EOG [41, 50]. The task consisted of 40 trials, each beginning with the presentation of a white fixation cross (1000 ms duration), followed by the simultaneous presentation of the two study cues on the left and right side of the screen (2000 ms). After cue offset, a small brown rectangle appeared behind one of the images with either a white circle or square visual probe inside the rectangle. Participants were told to identify the shape (circle or square) as quickly as possible by pressing one of two keyboard keys. After a response (or 10 s timeout without response), a variable inter-trial interval began (750–1250 ms) followed by the next trial. Within each trial, cue type and location (ocean/mountain, left/right), probe type (square/circle), and probe location (left/right) was counterbalanced across trials, and each combination was presented randomly. EOG recordings were obtained by first cleaning and exfoliating the skin, followed by placement of 4 mm Ag/AgC1 electrode pairs (filled with electrolyte gel) 1.5 cm from the outer canthus of each eye. For the ground electrode, an 8 mm Ag/AgC1 electrode was attached to the forehead. Impedance values were measured using a Checktrode (Model 1089 MK III, UFI, Morro Bay, CA), and electrodes with impedance values above 20 kΩ were reapplied. EOG signals were amplified, digitized, and sampled at 1000 Hz using an EOG100C amplifier (Biopac MP150 system) and AcqKnowledge software (Biopac, Goleta, CA). Trained raters excluded trials using the following criteria: 1) Eye gazes were not centrally fixated prior to the trial, 2) Initial eye gazes were < 100 ms after cue onset (indicating anticipatory eye-movements), and 3) Noise obscured direction of eye movements. Two primary outcome measures from this task were quantified using EOG-based eye-tracking, 1) First-gaze proportion, which was calculated as the direction of first gaze for each trial (towards the ocean or mountain image) when the cues appeared, as a fraction of total valid gazes (measuring initial attention), and 2) Mean gaze time spent looking at each cue (sustained attention). Note that the visual probe task used for the follow-up sessions included a short stimulus presentation (500 ms), which has been used previously to assess attentional bias through measurements of reaction time (RT) for discrimination of probe type as the primary outcome measure [51].

#### Emotional Reactivity (EMG)

Emotional reactivity was assessed by measuring corrugator and zygomatic reactivity in response to each cue [12, 13, 52]. During this task, completed at the pretest and post-test, each cue was presented ten times for 6000 ms in a random order. In between each cue presentation, a white fixation cross was presented for 4500–5000 ms. EMG activity was measured over left brow and cheek using 4 mm Ag/AgC1 electrodes and an 8 mm gel-filled Ag/AgC1 ground sensor on the forehead. Sites were cleaned with alcohol and lightly exfoliated and any site with impedance above 20 kΩ (measured with a Model 1089 MK III Checktrode, UFI, Morro Bay, CA) was reapplied. EMG signals were amplified, 10–500 Hz band pass filtered, digitized at 1000 Hz, 60 Hz band stop filtered, rectified and integrated over 20 ms by using EMG 100C amplifiers and MP150 Data Acquisition System and Acqknowledge software from Biopac Systems (Goleta, CA).

### Subjective Drug Effect Measures

Subjective drug effects were assessed during conditioning sessions using three measures. The Drug Effects Questionnaire (DEQ)[53] measures subjective drug effects and consists of five questions which ask participants to rate drug effects on a 100 mm visual analogue scale in terms of whether they “like,” “feel,” “dislike,” and “want more” of the drug, and how much they feel “high.” The Profile of Mood States (POMS)[54] measures subjective mood effects and is a 72 adjective list that asks participants to rate how much an adjective applies to them on a five point Likert scale, from 0 (“not at all”) to 4 (“extremely”). The primary outcome measures are eight clusters of items, termed scales (e.g., Anxiety, Depression, Anger, Vigor, Fatigue, Confusion, Friendliness, and Elation). The Addiction Research Center Inventory (ARCI)[44, 55] was used to assess general subjective drug effects and is a 52-item true/false scale with two subscales of interest, arousal (A) and euphoria (MBG).

### Cardiovascular Drug Effect Measures

HR and BP were monitored during the conditioning sessions at 6 regular time points (TPs) during the 4 h sessions. Mean arterial pressure (MAP) was calculated using the formula: [systolic BP + 2 x diastolic BP]/3.

### Statistical Analysis

All data were analyzed using the SPSS statistical software package (version 22, IBM, Chicago, IL). Physiological data (HR and MAP) were analyzed using raw data. Subjective drug effects (POMS, ARCI, DEQ) were analyzed using change scores, i.e., the difference between scores at each TP and the pre-drug score (i.e., TP 1). Session differences were determined for groups P2 and P4 using a repeated measures analysis of variance (rmANOVA) with Treatment (MA, PBO), Session (2 or 4), and Time (baseline, five TPs after drug administration) as within subjects factors. Group differences for change scores were assessed using average session data with Treatment and Time as within subjects factors and Group (P1, P2, P4) as a between subjects factor. Peak change scores (PCS) were calculated using the maximum change score over the course of the 6 TPs during conditioning sessions and were analyzed using a Group by Treatment by Session rmANOVA.

For analyses of behavioral preference, choices for the MA-paired cue were assessed at five sessions (before and after conditioning, and at three follow-ups 2, 7, and 20 d). A rmANOVA compared behavioral choice scores using a Group by Time analysis. VAS scores of cue “liking” were calculated using averaged scores for the two types of cue presentations (2 for MA and PBO each) at each of five sessions. Behavioral preference scores were also calculated by subtracting the PBO scores from MA scores at each TP. Analysis of VAS scores for the MA and PBO cues, and preference scores, were conducted using Group by Time rmANOVA analysis.

For attention measures, first-gaze proportions towards the MA-paired cue were calculated as the total number of initial gazes towards the MA-paired cue immediately after cue onset, as a fraction of total gazes (MA and PBO). A rmANOVA compared scores using Group by Time (before and after conditioning) analysis. Mean gaze times for each cue were averaged across 20 MA and PBO trials, before and after conditioning, and analyzed using a rmANOVA Group by Cue Type (MA/PBO) by Time analysis. Average RT data from the dot probe task were calculated for each probe type at the three follow-up sessions and a rmANOVA compared Group by Cue Type by Time. EMG data were analyzed for zygomatic and corrugator channels separately, using mean EMG responses for each of the 10 MA- and PBO-paired cue trials. Averaged data were analyzed using rmANOVA Cue Type by Time analysis. All planned post-hoc pairwise tests used the Bonferroni correction.

In separate analyses, we examined the relationship between the four outcome measures using Pearson’s product-moment correlations on change scores (before vs after conditioning) for each outcome measure. Using similar correlation analysis, acute responses to MA (vs PBO) and conditioned response measures were compared using averaged PCS for MA and PBO sessions. Similar correlation analyses compared the four outcome measures with the average inter-session interval for conditioning sessions for each individual.

## Results

### Participants

The characteristics of the participants are summarized in Table 1. The groups P1 (N = 13), P2 (N = 16), and P4 (N = 16) did not differ on any of the measures examined. Although about half the subjects reported having ever used stimulants in their lifetime, no participants reported prior use of methamphetamine (not shown in Table 1).

### Subjective and Cardiovascular Effects of Methamphetamine During Conditioning

Methamphetamine (MA; 20 mg) produced prototypic subjective effects (mood, ratings of drug effects) and alterations in physiology (heart rate, HR, blood pressure). The groups did not differ in these effects except on HR, where the P1 group exhibited a later increase than groups P2 and P4 (see below). Responses to MA compared to PBO did not differ across successive sessions in groups P2 and P4.

#### Cardiovascular Measures

MA significantly increased HR compared to PBO regardless of group [Treatment x Time interaction, F_(3.73, 156.62)_ = 56.40, *p* < 0.001, η_p_^2^ = 0.573, corrected for sphericity]. HR increased more quickly for groups P2 and P4 compared to P1. That is, at TP 3 HR was higher in the MA condition for groups P2 and P4 individually, but not group P1 (*p*’s = 0.017 and 0.022, respectively). At other time points the groups did not differ. Analysis of peak change scores (PCS) found similar results and post-hoc comparisons between treatments indicated treatment effects for all three groups (*p*’s < 0.001) and are listed in Table 2. MA significantly increased MAP compared to PBO [Treatment x Time interaction, F_(5, 210)_ = 46.49, *p* < 0.001, η_p_^2^ = 0.525], at TPs 3–6 for all three groups [all *p*’s ≤ 0.017], but the groups did not differ. Analyses of MAP PCS values yielded similar results (Table 2).

**Table 2:** Mean (SEM) Peak Change Scores (PCS) for Subjective Ratings and Cardiovascular Measures Averaged Across the Conditioning Sessions with Placebo or Methamphetamine.

		Group P1			Group P2			Group P4		
		Placebo	MA		Placebo	MA		Placebo	MA	
		Mean (±SEM)	Mean (±SEM)	P-value	Mean (±SEM)	Mean (±SEM)	P-value	Mean (±SEM)	Mean (±SEM)	P-value
**Subjective Effects**										
**DEQ**		N = 13	N = 13		N = 15	N = 15		N = 16	N = 16	
	Feel	21.70(7.24)	52.68(6.86)	<0.001***	15.62(4.11)	51.69(5.96)	<0.001***	11.68(3.17)	46.64(5.55)	<0.001***
	Like	30.96(7.27)	74.20(4.24)	<0.001***	18.90(4.30)	59.92(5.34)	<0.001***	12.73(3.32)	71.24(6.50)	<0.001***
	Dislike	29.51(9.11)	34.84(8.61)		26.74(7.66)	33.17(6.01)		25.92(6.74)	16.58(2.93)	
	High	16.69(6.94)	39.54(7.45)	0.001***	8.34(2.01)	42.87(6.81)	<0.001***	5.46(1.77)	38.53(6.12)	<0.001***
	More	18.04(6.36)	70.27(6.32)	<0.001***	12.93(3.80)	45.49(6.58)	<0.001***	10.60(3.50)	69.30(7.28)	<0.001***
**POMS**		N = 13	N = 13		N = 16	N = 16		N = 16	N = 16	
	Friendliness	0.38(1.62)	1.85(1.62)		-3.00(1.04)	0.63(0.58)	0.006**	-2.95(0.76)	3.48(1.27)	<0.001***
	Anxiety	-0.31(1.16)	0.85(1.25)		0.06(0.43)	2.34(0.62)	0.046*	0.63(0.31)	1.41(0.95)	
	Elation	-0.38(0.89)	2.77(1.36)	0.036*	-2.28(1.05)	1.44(0.88)	0.008**	-1.21(0.49)	4.19(0.92)	<0.001***
	Anger	-0.38(0.50)	-0.08(0.49)		0.06(0.29)	-0.25(0.41)		0.50(0.24)	-0.02(0.38)	
	Fatigue	0.85(1.17)	-0.23(1.01)		3.44(0.85)	-0.41(0.44)	0.001***	2.89(0.81)	-1.17(0.70)	<0.001***
	Confusion	-0.38(0.80)	1.31(0.82)		1.44(0.65)	0.50(0.53)		0.92(0.32)	-0.63(0.48)	
	Depression	-1.00(0.60)	0.92(1.35)		0.25(0.34)	0.00(0.22)		-0.63(0.17)	-0.39(0.31)	
	Vigor	-1.23(1.52)	3.00(2.62)	0.027*	-3.84(1.13)	2.69(1.86)	<0.001***	-1.28(0.69)	6.03(1.25)	<0.001***
**ARCI**		N = 13	N = 13		N = 16	N = 16		N = 16	N = 16	
	A	0.08(0.82)	2.85(0.93)	<0.001***	0.16(0.23)	3.28(0.59)	<0.001***	-0.06(0.36)	4.44(0.58)	<0.001***
	MBG	0.46(1.18)	4.15(1.64)	0.001***	-0.25(0.61)	4.25(0.76)	<0.001***	-0.28(0.64)	7.06(1.35)	<0.001***
**Cardiovascular**		N = 13	N = 13		N = 16	N = 16		N = 16	N = 16	
	BP	-3.79(2.55)	13.03(4.15)	<0.001***	-5.44(1.44)	12.79(2.36)	<0.001***	-8.03(1.75)	12.38(2.07)	<0.001***
	HR	-10.23(2.30)	7.15(4.60)	<0.001***	-4.59(2.90)	12.72(3.78)	<0.001***	-2.56(2.12)	17.19(2.80)	<0.001***

Abbreviations: DEQ, Drug Effects Questionnaire, POMS, Profile of Mood States, ARCI, Addiction Research Center Inventory. Blood pressure is represented as Mean Arterial Pressure. Note that all reported *p* values are derived from planned, within group, post-hoc pairwise comparisons between each treatment following rmANOVAs comparing Group x Treatment, and only for measures in which a significant main effect (Treatment) was found. No systematic Group x Treatment interactions were found.

* denotes *p* values < 0.05

** denotes *p* values < 0.01, and

*** denotes *p* values ≤ 0.001.

#### Subjective Measures

MA produced characteristic changes in subjective mood states, and these effects were similar across the groups. The effects of MA appeared to occur more quickly and last longer for groups P2 and P4 compared to group P1 for the two ARCI subscales (Arousal and Euphoria), and three subscales of the POMS (Friendliness, Elation and Vigor), but no significant group differences were found. PCS analyses of treatment effects for each group are listed in Table 2.

1. ARCI. MA increased mean ARCI Arousal (A) and Euphoria (MBG) subscale scores across 6 TPs [significant Treatment x Time interactions, F’s_(3.35, 137.21 and 3.39, 138.80)_ = 31.94, 26.99, *p*’s < 0.001, η_p_^2^’s = 0.437, 0.395, respectively, corrected for sphericity], starting at TP 3 [all *p*’s ≤ 0.019]. No group differences were observed. Note that two subjects were excluded from analyses (one from P1 and P4 groups) due to missing data.
2. POMS. MA increased mean POMS subscale scores of Friendliness, Elation, Vigor, Anxiety, and decreased Fatigue across 6 TPs [rmANOVA, significant Treatment x Time interactions, F’s_(3.22, 131.86), (2.92, 119.81), (2.81, 114.99), (2.89, 118.29), (3.08, 126.14)_ = 15.22, 16.70, 21.06, 4.66, 12.69, all p’s < 0.005, η_p_^2^’s = 0.271, 0.102, 0.289, 0.339, 0.236, respectively, corrected for sphericity], with significant differences starting at TP 3 [all p’s ≤ 0.045]. No significant effects were observed for measures of Anger, Confusion, or Depression. No group differences for any measure were found.
3. DEQ. MA increased mean scores on DEQ measures of “Feel,” “Like,” “Dislike,” “High,” and want “More” drug across 6 TPs [rmANOVA, significant Treatment x Time interactions, *Fs*_(3.44, 137.51), (3.20, 127.82), (2.61, 104.32), (2.64, 105.71), (2.60, 103.89)_ = 43.03, 36.95, 4.06, 30.55, 53.98, all *p*’s ≤ 0.012, η_p_^2^’s = 0.518, 0.480, 0.092, 0.433, 0.574, respectively, corrected for sphericity], with effects beginning at TP 2 or 3 and lasting until the end of the session [all *p*’s ≤ 0.043]. For “Dislike,” MA increased scores at TP 6 for group P1 [*p* = 0.013], had no significant treatment effects for group P2, and showed a marginally significant reduction compared to PBO for group P4 at TP 4 [*p* = 0.058]. No group differences were found for any measure.

### Conditioned Response Measures

#### Behavioral Preference

Behavioral preference choice scores for the MA-paired cue did not increase from before to after conditioning for any group, and no preference changes were evident during the three follow-up assessments (2, 7, and 20 d) (See S1 Table). Bias for distractor task images was also assessed, and although there was a tendency for subjects to prefer one task image over another, the change in preference from before to after conditioning did not systematically differ between the groups. Note that three subjects were removed from analyses for the forced choice task because they had near-maximal preferences for the MA or PBO cue at pre-test (one from group P2 and two from P4).

#### Self-reported “Liking”

**Fig 1:**
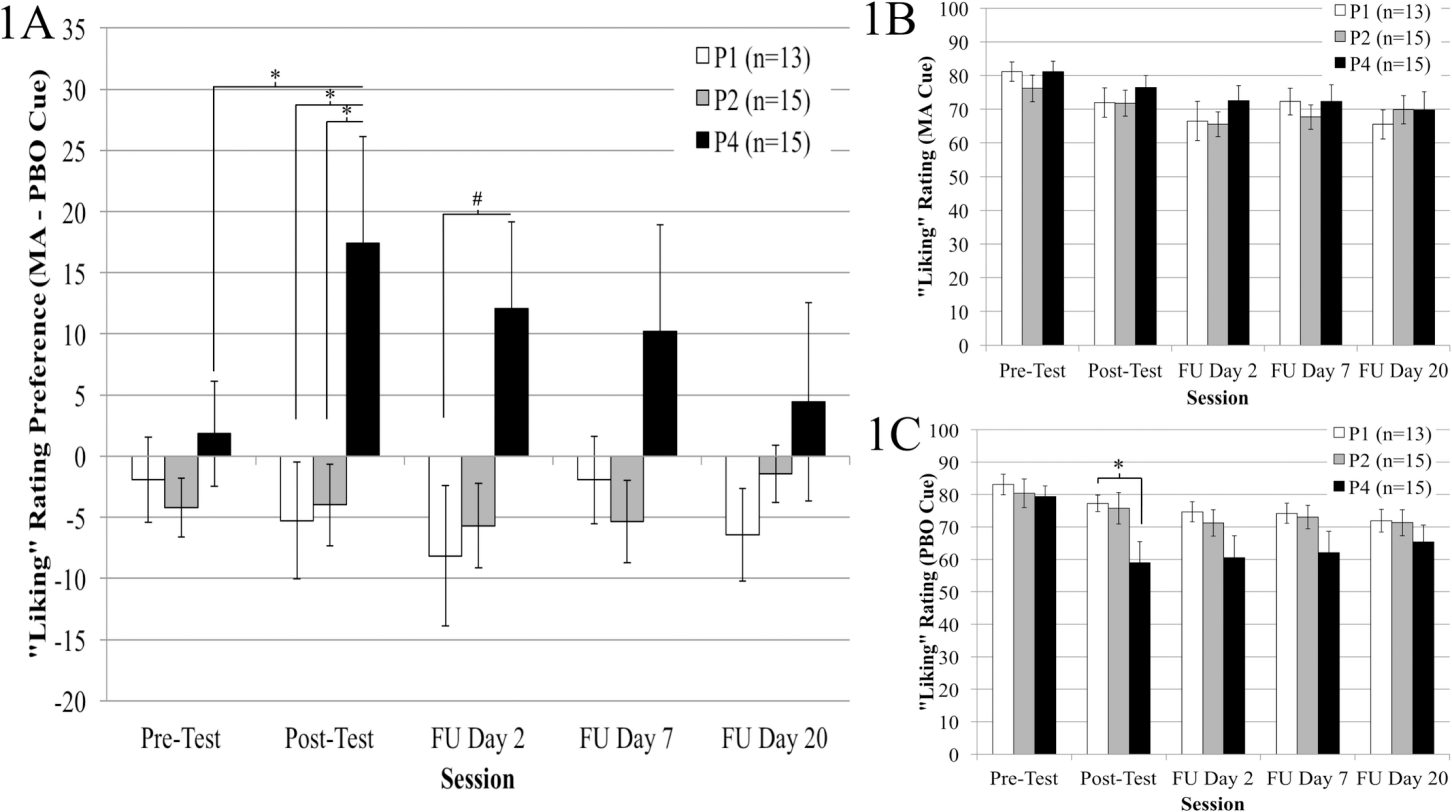
**Mean (± SEM) rating preference scores (A), and “liking” scores towards the MA- (B) or PBO-paired (C) stimuli before and after conditioning (Pre-/Post-Test), and at three follow-up sessions (FU) 2, 7, and 20 days after the post-test session.** Preference (A) scores (MA minus PBO “liking”) increased from before to after conditioning, but only for group P4. Preference scores for group P4 were significantly elevated compared to groups P1 and P2 at the post-test session. Two days after the post-test, group P4 showed marginally significant elevations compare to group P1 and P2. No significant changes in “liking” were observed for the MA-paired cue (B), while “liking” of the PBO-cue showed significant group differences after conditioning (C). At the post-test, PBO “liking” scores for group P4 were significantly less than group P1 and marginally less than group P2. Note: * denotes *p* values < 0.05, # values = 0.05.

Relative to PBO ratings, subjects in group P4 (but not P1 or P2) reported “liking” the MA-paired cue (VAS scores) more after conditioning (Fig 1A). For all three groups, ratings of both MA- and PBO-paired cues decreased after conditioning (rmANOVA, significant main effects of Time, F’s_(1, 40)_ = 9.83, 22.97, *p*’s ≤ 0.003, η_p_^2^’s = 0.197, 0.365) (Fig 1B and 1C, see S1 Table). However, ratings of “liking” of the MA cue declined less for group P4 (i.e., MA “liking” minus PBO “liking” preference). “Liking” preference ratings before and after conditioning were greater for group P4 (Mean ± SEM, 1.83 ± 4.30 and 17.43 ± 8.68, respectively) compared to groups P1 (-1.92 ± 3.50, -5.27 ± 4.80) and P2 (-4.23 ± 2.40, -4.0 ± 3.30) [significant Group x Time interaction, F_(2, 40)_ = 4.06, *p* = 0.025, η_p_^2^ = 0.169, significant pairwise comparison, *p* = 0.003].

During the follow up assessments 2, 7, and 20 days later, only the P4 group showed significantly increased “liking” preference ratings for the MA-paired cue over the three sessions (12.07 ± 7.08, 10.23 ± 8.67, 4.44 ± 8.11, respectively) compared to groups P1 (-8.15 ± 5.75, -1.94 ± 3.57, -6.44 ± 3.78) and P2 (-5.89 ± 3.46, -5.34 ± 3.37, -1.43 ± 2.34) [significant Group by Time interaction, F_(8, 160)_ = 2.15, *p* = 0.034, η_p_^2^ = 0.097] (Fig 1A). Group comparisons at each session found that at the 2 d follow-up, group P4 showed greater “liking” preference compared to group P1 (marginally significant, *p* = 0.05), and a trend elevation compared to group P2 (*p* = 0.084). No group differences were detected at the 7 and 20 d follow-up. Note that for all VAS analyses, data from two participants (1 each from groups P2 and P4) were excluded from analyses due to outlier status (i.e., preference rating scores before conditioning were > 2 standard deviations above/below mean values.

#### Attentional Bias

**Fig 2:**
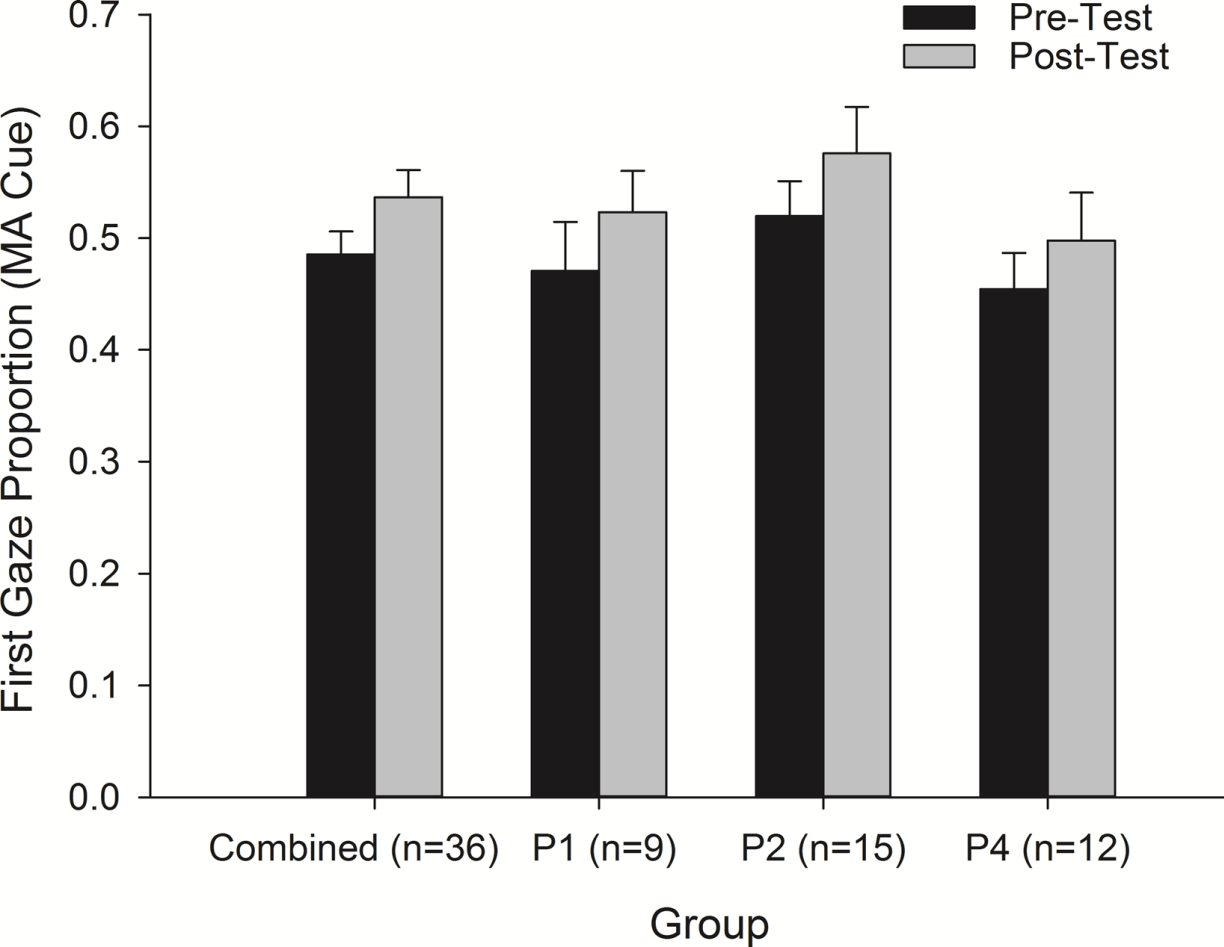
Mean (± SEM) first gazes towards MA-paired cue as a proportion of total gazes. Initial attention towards the drug-cue increased from before to after conditioning, collapsed across all groups. No group differences were found. Note: * denotes *p* value = 0.032.

The proportion of first gazes (i.e., initial attention bias) towards the MA-paired cues increased from before to after conditioning to a similar extent in all three groups, means ± SEM of all three groups combined were 0.49 ± 0.02 and 0.54 ± 0.02, respectively (N = 36) [rmANOVA, main effect of Time, F_(1, 33)_ = 4.99, *p* = 0.032, η_p_^2^ = 0.131] (Fig 2). Individual group analysis indicated no significant increases in gaze proportions before and after conditioning for groups P1 (0.47 ± 0.04, 0.52 ± 0.037, respectively), P2 (0.52 ± 0.03, 0.58 ± 0.04), and P4 (0.45 ± 0.03, 0.50 ± 0.04). However, gaze duration (i.e., sustained attention) did not change (see S1 Table). Note that for this task, we excluded participants who looked at the cues less than 50% of trials during the pre-conditioning test (e.g., four participants were excluded from groups P1 and P4 and one from group P2 for total N’s of 9, 15 and 12, for groups P1, P2 and P4, respectively). The dot-probe RT data at the three follow-up tests did not differ between cues or groups.

#### Emotional Reactivity

EMG activity for the zygomatic and corrugator muscles in response to the MA- and PBO-paired cues did not change after conditioning (see S1 Table).

#### Correlations between outcome measures

Changes in VAS preference “liking” ratings for the MA-paired cue (from before to after conditioning) were positively correlated with changes in behavioral preference scores on the forced choice task (Pearson’s *r* = 0.588, *p* < 0.001, N = 39). Within-group analyses indicated that this relationship was significant for group P2 (*r* = 0.790, *p* = 0.001, N = 14), marginally significant for group P4 (*r* = 0.574, *p* = 0.051, N = 12), but not for group P1. This suggests that individuals who displayed greater changes in behavioral preference for the MA-paired cue also exhibited stronger preference “liking” ratings for the MA cue, but this relationship only emerged after at least two MA-cue pairings. No significant correlations were observed for the changes in attentional or emotional reactivity measures.

#### Relationships between subjective drug effects and conditioned responses

**Fig 3:**
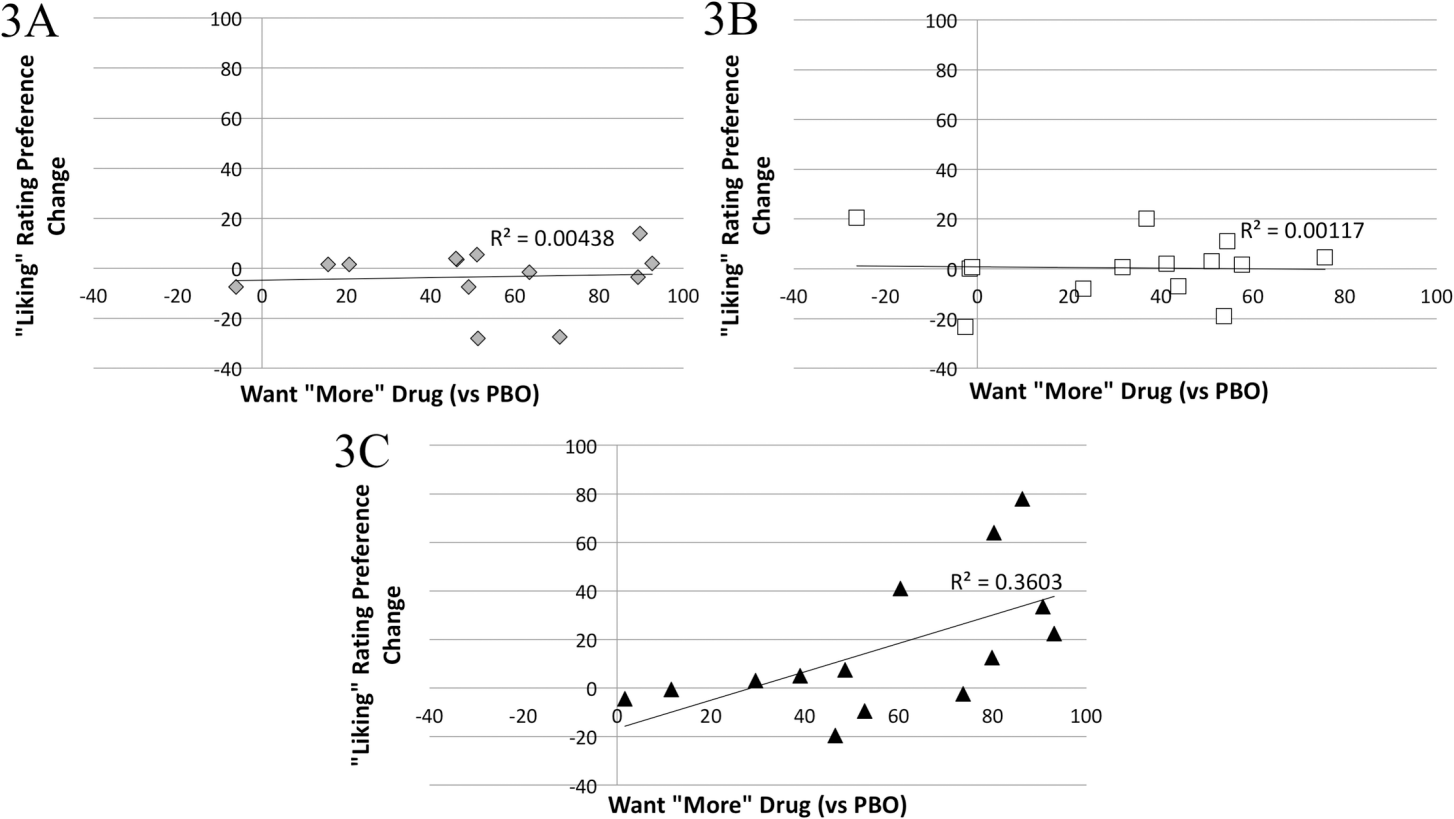
**Correlation between DEQ want “more” drug scores (MA minus PBO) during conditioning and VAS “liking” preference change scores (MA minus PBO, before vs after conditioning) towards the MA-paired cue for groups P1 (A), P2 (B), and P4 (C).** A significant positive correlation was found between want “more” and MA-cue “liking,” but only for group P4 (C, Pearson’s *r* = 0.600, *p* = 0.023).

Ratings of MA “Liking” (vs PBO) during conditioning (average PCS) were positively correlated with the change in VAS preference rating for the MA cue (Pearson’s *r* = 0.352, *p* = 0.024, N = 41). That is, when all 41 participants were examined together, those who reported greater MA liking showed the greatest changes in VAS preferences rating for the MA cue. Separate group analyses revealed no group differences. Ratings of wanting “More” MA (vs PBO, average PCS) during conditioning were also positively correlated with changes in VAS preference rating for the MA cue (*r* = 0.600, *p* = 0.023, N = 41) (Fig 3). When analyzed separately by group, this relationship was significant within group P4 (*r* = 0.600, *p* = 0.023, N = 14), but not for groups P1 and P2. This suggests that participants who reported wanting more MA exhibited greater changes in rating preference for the drug-paired cue, but this relationship only existed for those individuals who had received four drug-cue pairings. No other significant relationships were identified.

#### Relationship between inter-session interval and conditioned responses

The interval between conditioning sessions was unrelated to the conditioned responses on any of the four outcome measures.

## Discussion

The purpose of this study was to replicate and extend previous findings [3, 41] showing that healthy young adults acquired conditioned responses to a visual/auditory cue paired two times with methamphetamine. In the present study we expanded this procedure to compare the effects of one, two or four drug-cue pairings (groups P1, P2, and P4) testing the hypothesis that the magnitude of the conditioned-drug cue responses would increase monotonically with increasing number of drug-cue pairings. Our results only partially supported this hypothesis. The only clear difference in conditioned responses across the three groups was on ratings of “liking” of the cue: Subjects in the P4 group rated their “liking” of the MA-paired cue, related to PBO cue, higher after conditioning. All three groups exhibited an increase initial attention toward the drug-paired cue after conditioning.

The conditioned responses to the MA-paired cue were modest, especially relative to our previous studies using this procedure. For example, in the previous studies, we detected conditioned responses on behavioral preference and emotional reactivity after two pairings, but these measures did not increase in this study. The reasons for the differential results are not clear. It is possible that the apparently minor methodological changes, especially the different and increased number of distractor tasks, could have influenced the results. For example, one of the tasks used previously [56] was selected to activate reward pathways and involved monetary rewards. However, the tasks used in the present study also involve rewards (e.g., the BART and Gluck tasks). Alternatively, differences in the number or demographic characteristics of the participants might also have influenced the outcome. Whatever the reason, the fact that the conditioning indices were less robust than in the previous studies makes it difficult to draw firm conclusions about the relationship between number of pairings and conditioned responses using other outcome measures.

Conditioning studies with laboratory animals indicate that the magnitude and persistence of conditioned responses are directly related to the number of pairings between the conditioned stimulus (CS) and unconditioned stimulus (US) [57]. Notably, however, this relationship may not hold across all measures of conditioning. As noted above, conditioning can be measured in many ways, which may be dissociable and reflect separate underlying processes. Conditioned drug-cue responses may be assessed using physiological, behavioral as well as self-reported measures (see [10, 24, 58] for reviews). In our studies with humans, we have examined manifestations of the conditioned drug responses in the form of behavioral preference (i.e., preference for one stimulus over the other), attentional bias (i.e., tendency to look toward one stimulus), emotional reactivity as measured by electrophysiological indices of facial muscles (i.e., smile or frown), and subjective ratings of “liking” of the stimulus. These measures are not necessarily correlated with each other, and may be determined by different factors. In the present study, we saw little evidence for conditioning on two primary outcome measures (behavioral preference and emotional reactivity), even though the methods were similar to our previous study [41]. Conditioned increases in behavioral preference and emotional reactivity also did not develop when we tested this procedure with alcohol [59]. Nevertheless, we observed an increase in attentional bias for all subjects, and a relative increase in “liking” of the MA-paired stimulus among participants who experienced four pairings of MA and cue. Although it is not clear why the outcomes vary across studies, the dissociability among the measures suggests that distinct processes mediate the learning that occurs with different response modalities.

The relative increase in ratings of “liking” of the MA-paired cue in the P4 group followed an interesting pattern. That is, whereas subjects’ absolute ratings of the MA cue “liking” did not increase from before to after conditioning, their ratings of the PBO cue (but not the MA cue) declined after conditioning. This is similar to our previous study [41], in which “liking” for both cues (MA and PBO) decreased after conditioning. It is possible that participants rate the images as less positive after conditioning because they lose their novelty. It is noteworthy that the decline in “liking” ratings in the present study was greatest after more sessions and exposures (i.e., in group P4), consistent with an interpretation of habituation or boredom with the stimuli. Looked at this way, MA attenuated the decline in “liking” of the cue, perhaps because the cue acquired positive hedonic properties. In interpreting this trend, it is noteworthy that participants were told they might receive a stimulant, a tranquilizer or placebo, and thus they might have believed the placebo was an active drug, thereby forming a negative association between the placebo session and background cue. Indeed, informal post-hoc analyses indicated that a relatively high proportion of participants (19/45) incorrectly labeled the placebo as sedative, and these subjects also “disliked” what they received on those sessions (i.e., they rated it less than 50, neutral, on a 0–100 mm scale). Interestingly, we observed a similar, paradoxically negative reaction to PBO in our previous study [41], in which the MA-paired cue increased zygomatic (smile) activity whereas the PBO cue significantly decreased zygomatic activity. It remains to be determined whether the negative response to the PBO cue is a result of the repeated exposure to the stimulus itself, or whether it is the result of a contrast to the active drug condition.

Despite the absence of a significant increase in behavioral preference (forced choice) after conditioning, subjects’ rated “liking” for the drug-related stimulus was positively correlated with behavioral cue preference within the P4 group. This suggests that with a larger sample it may be possible to demonstrate conditioned effects with the behavioral preference measure. Alternatively, it is also possible that behavioral indices of “seeking” (i.e., behavioral preference) are separate from measures of “liking” of a drug or stimulus (see [60] for review). The present findings reinforce the idea that conditioning processes are complex: Conditioned responses may take different forms (e.g., behavioral, subjective), they can vary across individuals, and in our procedure the conditions that control the acquisition of conditioned responses are poorly understood.

The present findings can be compared to previous studies, without regard to the number of trials. As noted above, we previously reported that MA-cue pairings increased behavioral preference, emotional reactivity, and attentional bias as measured by sustained attention (mean gaze time) towards the drug-paired cue, whereas here we observed only increased attentional bias as measured by initial gaze and cue “liking.” The reasons for the differences are not known, but could be related to differences in sample size (N = 90 vs N = 42). On the measure of behavioral preference, Mayo and de Wit (2015) found that preference increased in 50/90 participants (55.5%) compared to the 19/42 (45.2%) participants in the current study. As noted above, subject sampling and methodological differences might also have contributed in ways that are difficult to detect. For example, when alcohol was used as the drug reinforcer, Mayo and de Wit (2016) observed increases in initial gazes towards the drug-paired cue, but no changes in emotional reactivity, possibly indicating that participant sampling influences the specific expression of conditioned drug-cue responses.

Limitations of this study include a small sample size and the use of healthy participants. As noted above, the failure to replicate some of our previous findings may be related to the small number of subjects, and relatedly, more subjects may be needed to detect the effect of number of pairings. The participants in this study were healthy adults with no history of problem drug use. It is possible that this approach under-sampled individuals with a predisposition for drug addiction or who were sensitive to drug-cue conditioning.

In conclusion, results from laboratory models of drug conditioning, such as this study, advance our basic understanding of how conditioned drug-cue responses are acquired [1, 2, 37, 38, 61]. In this study, we examined how the number of stimulus-drug pairing affected the magnitude of conditioned responses, using a tightly controlled *de novo* conditioning paradigm with discrete neutral cues paired with methamphetamine. Our findings provide some evidence that conditioned responses (i.e., “liking” of the cue) are greater with more pairings. The findings also provide preliminary evidence on individual differences in susceptibility to developing conditioned drug-cue responses based on the relation between individual subjective responses to a drug. New treatment strategies might utilize this information and target drug users who are particularly sensitive to developing conditioned drug responses, and targeting the cue-induced response using extinction or exposure therapy [62]. Future studies like this will furnish insight into the processes underlying conditioned drug effects and their role in future drug-seeking.

## Supporting Information

S1 Table Mean values (± SEM) for primary outcome measures after 1, 2, or 4 pairings with methamphetamine (groups P1, P2, and P4), before and after conditioning. Note that cue types indicate whether the cue was paired with methamphetamine (MA) or placebo (PBO).
(DOCX)
