# Supplementary material for: Acquisition of Conditioning between Methamphetamine and Cues in Healthy Humans
Source: PLoS One. 2016 Aug 22;11(8):e0161541. doi: 10.1371/journal.pone.0161541 (PMC4993385; doi:10.1371/journal.pone.0161541)
Supplement: S1 Table — Note that cue types indicate whether the cue was paired with methamphetamine (MA) or placebo (PBO). (DOCX) [file pone.0161541.s001.docx]

Supplemental Table 1. Scores for Primary Outcome Measures, Mean (SEM)

| **Measure** | **Group** | **Session:** | **Pre-Test** | | **Post-Test** | | **Follow-Up 1** | | **Follow-Up 2** | | **Follow-Up 3** | |
| --- | --- | --- | --- | --- | --- | --- | --- | --- | --- | --- | --- | --- |
| Choice Preference |  | |  | |  | |  | |  | |  | |
|  | P1 (N=13) |  | -0.31(1.72) | | -0.92(2.69) | | -1.54(2.26) | | -2.69(2.44) | | 0.31(2.43) | |
|  | P2 (N=15) |  | 0.13(1.55) | | -0.27(1.92) | | 1.47(1.65) | | 1.40(1.90) | | -0.13(1.87) | |
|  | P4 (N=14) |  | -2.14(1.76) | | 0.14(2.56) | | 1.14(2.53) | | 0.86(2.47) | | 0.29(2.73) | |
|  |  |  |  |  |  |  |  |  |  |  |  |  |
| **Measure** | **Group** | **Session:** | **Pre-Test** | | **Post-Test** | | **Follow-Up 1** | | **Follow-Up 2** | | **Follow-Up 3** | |
|  |  | Cue Type: | MA | PBO | MA | PBO | MA | PBO | MA | PBO | MA | PBO |
| “Liking” |  | |  |  |  |  |  |  |  |  |  |  |
|  | P1 (N=13) |  | 81.2(2.9) | 83.1(3.1) | 72.0(4.4) | 77.3(2.6) | 66.5(5.8) | 74.7(3.1) | 72.3(4.0) | 74.2(3.1) | 65.5(4.3) | 71.2(3.5) |
|  | P2 (N=15) |  | 76.2(4.0) | 80.4(4.4) | 71.8(3.9) | 75.8(4.8) | 65.5(3.6) | 71.2(4.1) | 67.7(3.6) | 73.0(3.6) | 69.9(4.2) | 71.3(4.0) |
|  | P4 (N=15) |  | 81.2(3.1) | 79.3(3.3) | 76.4(3.5) | 59.0(6.4) | 72.6(4.4) | 60.5(6.8) | 72.4(4.9) | 62.1(6.5) | 69.8(5.3) | 65.4(5.2) |
|  |  |  |  |  |  |  |  |  |  |  |  |  |
| EMG Zyg |  |  |  |  |  |  |  |  |  |  |  |  |
|  | P1 (N=13) |  | 0.13(0.0) | 0.18(0.1) | 0.16(0.1) | 0.10(0.0) |  |  |  |  |  |  |
|  | P2 (N=16) |  | 0.18(0.1) | 0.13(0.1) | 0.15(0.1) | 0.05(0.0) |  |  |  |  |  |  |
|  | P4 (N=16) |  | 0.13(0.1) | 0.28(0.1) | 0.21(0.1) | 0.09(0.1) |  |  |  |  |  |  |
| EMG Cor |  |  |  |  |  |  |  |  |  |  |  |  |
|  | P1 (N=13) |  | 0.20(0.2) | -0.11(0.3) | 0.44(0.2) | 0.21(0.1) |  |  |  |  |  |  |
|  | P2 (N=16) |  | 0.21(0.2) | 0.07(0.1) | -0.02(0.2) | -0.28(0.1) |  |  |  |  |  |  |
|  | P4 (N=16) |  | -0.09(-0.2) | -0.10(0.2) | 0.13(0.2) | -0.15(0.2) |  |  |  |  |  |  |
|  |  |  |  |  |  |  |  |  |  |  |  |  |
| EOG |  |  |  |  |  |  |  |  |  |  |  |  |
|  | P1 (N=9) |  | 820(80) | 652(90) | 731(92) | 629(85) |  |  |  |  |  |  |
|  | P2 (N=15) |  | 480(64) | 586(74) | 494(64) | 580(99) |  |  |  |  |  |  |
|  | P4 (N=12) |  | 544(102) | 375(69) | 622(108) | 483(83) |  |  |  |  |  |  |

Mean values (± SEM) for primary outcome measures after 1, 2, or 4 pairings with methamphetamine (groups P1, P2, and P4), before and after conditioning. Note that cue types indicate whether the cue was paired with methamphetamine (MA) or placebo (PBO). EMG Zyg and EMG Cor labels refer to average values from zygomatic and corrugator channels, respectively. EOG values displayed are average gaze times in ms.
